# The role of chromatin remodeler *SMARCA4*/BRG1 in brain cancers: a potential therapeutic target

**DOI:** 10.1038/s41388-023-02773-9

**Published:** 2023-07-11

**Authors:** Sophie M. Navickas, Katherine A. Giles, Kate H. Brettingham-Moore, Phillippa C. Taberlay

**Affiliations:** 1 Tasmanian School of Medicine, College of Health and Medicine, University of Tasmania, 17 Liverpool Street, Hobart, TAS 7000 Australia; 2 Children’s Medical Research Institute, 214 Hawkesbury Road, Westmead, NSW 2145 Australia

**Keywords:** Cancer genetics, CNS cancer, Chromatin remodelling

## Abstract

The chromatin remodeler *SMARCA4*/BRG1 is a key epigenetic regulator with diverse roles in coordinating the molecular programs that underlie brain tumour development. BRG1 function in brain cancer is largely specific to the tumour type and varies further between tumour subtypes, highlighting its complexity. Altered *SMARCA4* expression has been linked to medulloblastoma, low-grade gliomas such as oligodendroglioma, high-grade gliomas such as glioblastoma and atypical/teratoid rhabdoid tumours. *SMARCA4* mutations in brain cancer predominantly occur in the crucial catalytic ATPase domain, which is associated with tumour suppressor activity. However, *SMARCA4* is opposingly seen to promote tumourigenesis in the absence of mutation and through overexpression in other brain tumours. This review explores the multifaceted interaction between *SMARCA4* and various brain cancer types, highlighting its roles in tumour pathogenesis, the pathways it regulates, and the advances that have been made in understanding the functional relevance of mutations. We discuss developments made in targeting *SMARCA4* and the potential to translate these to adjuvant therapies able to enhance current methods of brain cancer treatment.

## SMARCA4 as an epigenetic regulator in cancer

Epigenetic regulation is a crucial moderator of gene expression programs that underlie normal cellular function. Epigenetics refers to heritable changes that modulate gene expression without altering the DNA sequence [1]. Cancer is a disease driven by aberrant activity of signaling pathways, thus epigenetic regulation plays a major role in controlling the functional changes that occur in malignant cell transformation [2]. A degree of hidden variation exists within cancer that cannot be explained by genetic alterations alone, and epigenetic alterations are likely to account for this [3]. Driver mutations that give cancer cells a growth advantage have been frequently located in epigenetic regulator genes [4]. This is particularly relevant to brain cancers as many occur predominantly in paediatric patients and thus lack the considerable number of passenger mutations that are accumulated as a natural result of aging [5].

**Fig. 1:**
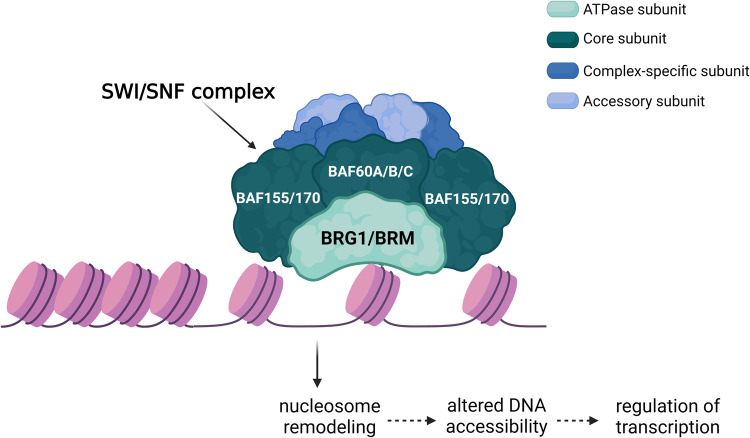
The SWI/SNF chromatin remodeling complex regulates gene expression. The core subunits of the SWI/SNF complex include ATPase subunit BRG1 *(SMARCA4*) or BRM (*SMARCA2*), BAF155/BAF170 (*SMARCC1/2*) and BAF60A/B/C (*SMARCD1/2/3*). BRG1 or BRM facilitate nucleosome remodeling by using energy from ATP hydrolysis to alter chromatin accessibility. The incorporation of complex-specific and accessory subunits varies according to the unique SWI/SNF complex configurations of BAF, PBAF and ncBAF. Created with BioRender.com.

ATP-dependent chromatin remodeling is an important epigenetic mechanism that regulates gene expression by controlling the dynamic and highly organised state of chromatin [6]. Altered expression of chromatin remodeler proteins is a common pan-cancer theme [7, 8]. The switching defective/sucrose non-fermentable (SWI/SNF) complex is a well characterised chromatin remodeling complex that is mutated in approximately 20% of human tumours, which is comparable to the mutation pattern of the familiar tumour suppressor gene *TP53* [9]. There are three classes of mammalian SWI/SNF complexes that differ in subunit composition, genome localisation, and have non-redundant functions. These are canonical BRG1/BRM-associated factor (BAF), polybromo-associated BAF (PBAF), and the more recently defined non-canonical (ncBAF), also known as GBAF due to the unique inclusion of the GLTSCR1 subunit [10, 11]. All SWI/SNF complexes assemble around an initial core that contains a dimer made up of BAF155/BAF170 (gene name *SMARCC1/2*), and one BAF60A/B/C subunit (*SMARCD1/2/3*), before complex-specific subunits are incorporated [10] (Fig. 1). The ATPase subunit, either BRG1 (*SMARCA4*) or BRM (*SMARCA2*), binds to the core BAF module and recruits accessory subunits to finalise SWI/SNF complex formation [10, 12]. One of the most frequently mutated subunits of the SWI/SNF complex in human cancers is *SMARCA4*, which encodes the BRG1 protein [9]. BRG1 utilises energy from ATP hydrolysis to physically rearrange nucleosomes and alter chromatin accessibility, making BRG1 function a central component in orchestrating cancer gene expression programs [7] (Fig. 1).

In addition to transcription, BRG1 also has roles in DNA processes which further contribute to its importance in cancer [13–15]. BRG1 has been shown to promote DNA repair at double-stranded breaks through nucleosome repositioning and recruitment of repair factors [13–15], whilst its absence has been demonstrated to induce replication stress which is a major cause of genome instability [16, 17]. The associations between BRG1 and cancer, and the types of genetic alterations that are observed are highly dependent on the cancer type. Multiple pan-cancer studies have documented *SMARCA4* genetic alterations from human tumour sequencing data, with a wide variety of aberrations observed [18–21]. BRG1 is commonly described to have a tumour suppressing role in cancers such as lung, ovarian, skin, and lymphoma [22–25]. Conversely, BRG1 has been implicated in coordinating and maintaining key signaling pathways that promote oncogenesis in other cancer types including leukemia, breast, and prostate cancer [26–29]. In brain cancer, BRG1 exhibits both tumour suppressor and oncogenic functions [30–33]. Whilst it is well documented that *SMARCA4* is frequently mutated in cancers, the functional consequences of specific *SMARCA4* mutations on cell physiology are poorly understood and lack useful application in a clinical setting.

This review discusses the current understanding of the role BRG1 plays in brain cancers and the differences that exist between brain cancer types. We outline the existing knowledge of *SMARCA4* clinical mutations, functional consequences of mutation, and recent advances in targeting *SMARCA4* as a potential therapeutic strategy.

## The role of SMARCA4 in brain cancer

Central nervous system (CNS) tumours are a major cause of cancer death worldwide, with the large majority of CNS tumours occurring in the brain [34]. CNS tumours are the most common solid tumour in infants and children [35]. The mortality rate and years of life lost due to cancer death associated with brain cancers is considerably greater compared to other cancer types due to limited treatment options and severe side-effects that can be detrimental to quality of life [35]. *SMARCA4* is a recurrently mutated gene in multiple types of brain cancer including medulloblastoma, glioma, and atypical teratoid/rhabdoid tumours [31–33, 36]. The role *SMARCA4* plays in tumourigenesis is highly variable and largely dependent on the type of brain tumour, with the *SMARCA4* mutational landscape across brain tumours being diverse [30, 32, 33, 36, 37]. Heterogeneity also exists within tumour types, with differing roles reported at a subgroup level [30, 32, 38].

## Brain cancers

### Medulloblastoma

**Fig. 2:**
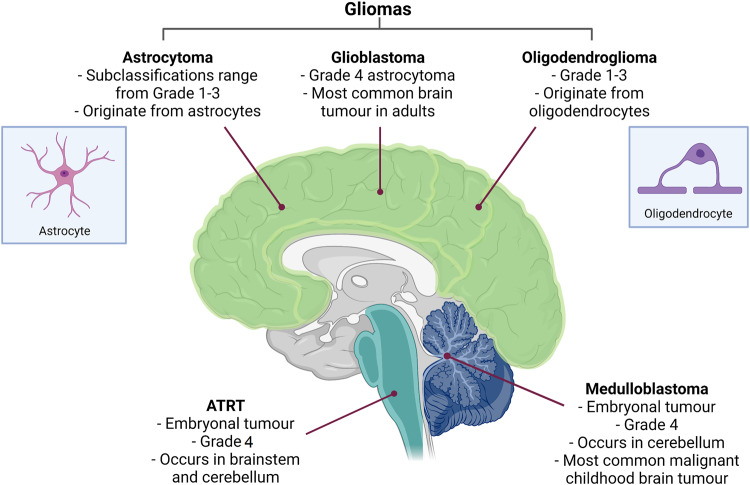
Summary of brain tumours where *SMARCA4* is implicated in pathogenesis. Brain tumour name, tumour classification and cell or histological site of origin. Classifications are based on the updated WHO Classification of Tumours of the CNS [41]. Grade 1–2 are low-grade tumours and grade 3–4 are high-grade tumours. Created with BioRender.com and adapted from Mulcahy et al. 2020 [107].

Medulloblastoma (MB) is the most common paediatric malignant brain tumour and accounts for around 10% of all childhood brain cancer cases [39, 40]. MB tumours are embryonal tumours that originate in the posterior fossa near the cerebellum [39] (Fig. 2). MB tumours grow rapidly and have high rates of metastasis via cerebrospinal fluid, therefore are classified by the World Health Organisation (WHO) as CNS WHO grade 4 tumours; the highest possible classification [41]. MB is primarily a paediatric disease, but it can occur in adults, albeit with generally lower risk of severe disease and better prognosis [42]. Severe adverse effects impacting neurocognitive and endocrine function are commonly associated with current standard MB treatments including surgery, chemotherapy, and radiotherapy [43]. This has a significant negative impact on paediatric patients where treatment often overlaps with crucial periods of brain development, highlighting a need for improved and more targeted therapies [39].

MB can be categorised into four molecular subgroups based on transcriptional and epigenetic profiles, which are Wingless (WNT), Sonic hedgehog (SHH), Group 3 and Group 4 [41, 44]. More recent studies have discovered that these principal groups can be further stratified within groups, with an important distinction made for SHH group tumours based on *TP53* status as *TP53*-mutant patients exhibit significantly poorer prognosis compared to *TP53*-wild-type patients [45, 46]. MB tumour subgroups display distinct age distributions, histology and have clear prognostic and clinical significance [47]. There is substantial evidence that supports *SMARCA4* having a critical role in MB as it has been identified as one of the few recurrently mutated genes [30, 36, 38]. Notably, *SMARCA4* mutations appear to be restricted to WNT and Group 3 tumours [31, 38, 48], and are rarely observed in SHH and Group 4 MB tumours [30, 49]. This indicates that BRG1 plays a different role within these subgroups.

*SMARCA4* mutation is common in WNT and Group 3 tumours. *SMARCA4* is mutated in around 26% of all WNT tumours and 11% of all Group 3 tumours, with some studies identifying *SMARCA4* as the most frequently mutated gene in Group 3 tumours [31, 38]. These two subgroups display very different prognoses, with the WNT subgroup having the best prognosis and a 5-year survival rate of 95%, compared to Group 3 MB which has the lowest overall survival of all MB subgroups at less than 50% [50]. Despite this discrepancy, *SMARCA4* appears to have a classic tumour supressing role in both subgroups due to the significant occurrence of characteristic inactivating point mutations associated with cancer development [36, 48]. Consistent with other paediatric cancers, MB has a relatively low mutation rate, emphasising the significance of the few recurrent mutations that occur in tumours [36]. *SMARCA4* mutations in WNT and Group 3 subgroups are almost exclusively missense mutations located in the functionally crucial ATPase domain and are commonly heterozygous [31, 36, 48]. Despite Group 4 being the most common MB subgroup, there is very little known about the involvement of *SMARCA4* in this group, except that mutation is rare and occurs at a rate comparable to SHH MB [43, 49]. This indicates that the role of *SMARCA4* in Group 4 could be neutral or alternatively it could potentially resemble its oncogenic function in the SHH subgroup, though this is yet to be widely explored. The major hinderance to *SMARCA4* functional studies in Group 3 and Group 4 in particular is the lack of knowledge about which genetic pathways underlie these groups meaning accurate in vivo models are limited [43].

*SMARCA4* is essentially free from mutation in the SHH subgroup of MB, in stark contrast to the high mutation rate observed in other subtypes [49]. One possibility is that *SMARCA4* is important for tumour viability in the SHH subgroup of MB. In support of this, it has previously been demonstrated that RNAi-mediated inhibition of BRG1 impaired cell growth and decreased expression of subtype specific oncogenes in the Daoy cell line, representative of SHH-group MB [30]. This was in comparison to the Group 3/4-like cell line D283, where BRG1 inhibition had no effect on cell growth [30]. In vivo studies have shown similar results, with BRG1 required for SHH-target gene expression and tumour cell proliferation in mice [51]. BRG1 deletion in the cerebellum of mice led to decreased expression of SHH-target genes and reduced proliferation of tumour cells [52]. Therapeutic targeting of oncogenes is commonly through small molecule inhibitors [53]. Thus, this strategy could be used to target the oncogenic effects of BRG1 in SHH MB. An important additional effect of BRG1 deletion was that it resulted in a smaller cerebellum in mice [52], suggesting that whilst *SMARCA4* may promote oncogenesis, it may also be required for normal cerebral development. Recent studies assessing the impact of BRG1 knockout in cerebellar granule neuron precursor cells (CGNPs), the cells of origin of SHH MB tumours, revealed that BRG1 knockout in mice CGNPs did not cause tumour development but rather resulted in severe CNS abnormalities [54, 55]. This indicates that there may be a temporal component to BRG1 function in CGNPs and that a crucial role in cerebral development precedes its aberrant function in tumour cell proliferation. Overall, the consensus of the literature is that *SMARCA4* supports SHH MB, therefore selective and timely inhibition of *SMARCA4* could be a potential therapeutic strategy to treat this tumour type. The degree to which the major oncogenic effects of BRG1 are through direct activity or indirect regulation of transcription of other oncogenes is yet to be determined. This is an emerging area of research and whilst currently no inhibitors of BRG1 have been approved for clinical use, an inhibitor that targets the ATPase domain of BRG1 has recently been validated for research purposes, which we discuss later [56].

### Glioma

Gliomas are the most prevalent primary tumours of the CNS [57]. Gliomas are divided into 6 different families by the WHO classification system, but broadly gliomas encompass astrocytomas, oligoastrocytomas, oligodendrogliomas, ependymal tumours and mixed neuronal tumours [41, 44, 57]. More comprehensive delineations exist within these groups based on histopathological features, including the classification of glioblastoma (GBM) as a high-grade astrocytoma [41, 57] (Fig. 2). An important distinction in glioma classification is the separation of adult-type and paediatric-type tumours, as these have markedly different molecular profiles and clinical implications [41]. Adult gliomas are much more likely to progress from low-grade to high-grade tumours compared to paediatric cases [40]. Despite recent molecular advances which have improved the accuracy of diagnosis and treatment, glioma prognosis remains poor for certain groups. 5-year survival rate for low-grade gliomas can be as high as 90%, whereas survival rate significantly drops for high-grade gliomas to as low as 7% [58].

### Glioblastoma

GBM has been the sole focus of numerous studies, prompted by an effort to improve the dismal prognosis and treatment resistance that is associated with this glioma subtype. GBM is defined as a high-grade malignant glioma of the astrocytic lineage and is classified as a CNS WHO grade 4 tumour [40, 41] (Fig. 2). GBM is the most common malignant brain tumour in adults, with incidence thought to peak around 75-years [59, 60]. GBM is much rarer in paediatric cases and has a slightly better survival rate compared to adult GBM, but both carry very poor prognoses with an average survival rate of less than 2 years post-diagnosis [40, 61]. The discrepancies in incidence and prognosis of the two groups may be partially attributed to the substantial molecular differences that exist between adult and paediatric GBM tumours. DNA copy number differences such as frequent gain of chromosome 1q in paediatric GBM and chromosome 7 in adult GBM distinguish the two groups, as well as mutational signatures such as *IDH1* that appear to be restricted to adult GBM tumours [62].

A significant increase in BRG1 expression has previously been reported in human GBM tumours [61, 63, 64]. This increase was consistently observed in patient biopsy samples and online patient databases, with GBM tumour tissue having higher BRG1 expression in comparison to both adjacent normal brain tissue and low-grade glioma [63, 64]. A recent review reported that paediatric high-grade gliomas such as GBM do not experience genetic alterations of any SWI/SNF genes [37]. Previous studies have indicated that *SMARCA4* mutations do occur in GBM but infrequently, with evaluation of the online cBioPortal database (https://www.cbioportal.org/) revealing that *SMARAC4* mutations are observed in less than 2% of GBM cases [20, 64]. In cases where there is an absence of a genetic mutation, there is potential for epigenetic regulation to be the driving factor of altered BRG1 expression. However, this is yet to be widely explored in the case of increased BRG1 expression which remains a major feature of GBM tumours.

A direct effect of increased BRG1 expression on GBM tumour cell proliferation, invasion and migration potential has been observed in vitro [63]. This was demonstrated in human GBM cell lines U251 and U87, where knockdown of BRG1 by siRNA caused G1 phase cell cycle arrest via downregulation of cyclin D1 and consequently inhibited cell growth [63]. Migration and invasion ability of glioma cells was also decreased following BRG1 knockdown, assessed by cell migration and Matrigel invasion assay, largely through the downregulation of MMP-2 expression [63]. This provides vital functional evidence that BRG1 is likely involved in promoting the tumourigenic properties of cell invasion and migration that make GBM such an aggressive type of glioma. CRISPR/Cas9 gene editing has been used to generate BRG1 knockouts (KO) in GBM cell lines MT330 and LN229 [64]. Both GBM cell migration and invasion were again significantly reduced following BRG1-KO, with a slight decrease in cell proliferation also observed [64]. Moreover, the effect of a common chemotherapeutic agent, temozolomide (TMZ), was enhanced at multiple doses post BRG1-KO [64]. This finding could improve efficacy of existing therapeutics if the use of BRG1 inhibitors can be translated into a clinical setting as adjuvant therapy. Analysis of gene expression changes showed that BRG1-KO downregulated the STAT3 pathway [64]. Constitutive activation of the STAT3 pathway in cancer has previously been implicated in promoting tumour proliferation, invasion, and metastasis [65]. Thus, interaction between BRG1 and the STAT3 pathway may be part of the mechanism by which BRG1 increases GBM tumour aggressiveness.

### Glioblastoma stem-cells and BRG1

Substantial evidence now implicates BRG1 in the maintenance the stem-like state of glioblastoma initiating cells (GICs) [59, 61]. GICs have the ability to self-renew, with this characteristic stimulating the development of tumour heterogeneity and cell populations that may be highly resistant to treatment [61]. The average time for GBM recurrence after surgical resection is 7 months and almost all GBM tumours eventually relapse [66]. Thus, the cause of such rapid recurrence poses a significant barrier to successful GBM treatment. Studies have shown that BRG1 is expressed at high levels in both patient-derived and cultured GICs [59, 61]. BRG1 knockdown in GICs in vitro has been demonstrated to decrease the expression of pluripotency markers and increase expression of differentiation markers, thus linking BRG1 to the maintenance of GIC stemness [59, 61]. It was further demonstrated that BRG1 regulates glycolysis-related genes necessary for GIC survival through a STAT3-dependent pathway [61]. This suggests that BRG1 knockdown would be beneficial in controlling unwanted tumour heterogeneity. However, BRG1 knockdown in GICs also caused an increase in cell proliferation compared to control cells, and in vivo exploration found that larger intracranial tumours were formed in BRG1 knockdown GIC-derived mouse models compared to mice where BRG1 expression was normal [61]. These findings conversely suggest that BRG1 restricts GIC proliferation in a beneficial way. However, it was noted that chemotherapeutic drugs preferentially target dividing cancer cells and that tumours were more differentiated [61]. A promising and clinically relevant finding was that reducing BRG1 expression sensitised both GICs and differentiated GBM tumour cells to the chemotherapeutic agent TMZ, likely due to the role BRG1 has in promoting DNA repair [61, 64]. Whilst BRG1 has a strong link to GBM, these findings highlight a level of complexity and uncertainty as to the exact mechanisms, with multiple roles described. Nonetheless, it highlights BRG1 and the molecular pathways it regulates, particularly the STAT3 pathway, as important factors to understand in GBM tumour aggressiveness.

### Oligodendroglioma

Oligodendroglioma grading is based on tumour growth rate and can vary in severity between grade 1 and 3 [67]. Multiple studies have reported recurrent *SMARCA4* mutations in oligodendroglioma from patient data [67–71]. These mutations are most commonly in the ATPase domain and mirror those observed in WNT and Group 3 MB subgroups [67–71]. A 2013 study investigated the association of various genetic variants of *SMARCA4* and *SMARCA2* with the risk of glioma subtype and mortality [32]. The study included adult patients with low-grade astrocytoma, oligodendroglioma and GBM. Overall, there was no association found between the SNPs investigated and general glioma risk [32]. However, when risk was assessed based on histological subtype, specific variants in *SMARCA4* and *SMARCA2* were correlated with a modest increase in risk of oligodendroglioma, but not astrocytoma or GBM [32]. Both variants of *SMARCA4* and *SMARCA2* were intronic and their exact functional impact is unknown [32]. However, intronic variants can disrupt functional RNA production and gene regulatory regions such as enhancers, potentially leading to aberrant gene expression [72, 73]. Though this finding is indirect and lacks a clear mechanism, it provides evidence that *SMARCA4* may play an important regulatory role in oligodendroglioma tumourigenesis. Due to the variable nature of oligodendroglioma tumour grade and aggressiveness, it is often grouped under the broader classification of glioma for research purposes and is a lesser focus of functional studies.

### Atypical teratoid/rhabdoid tumour

Atypical teratoid/rhabdoid tumours (ATRTs) are tumours of the CNS that predominantly occur in young children. The age of tumour presentation is typically before 5 years-of-age [74]. They are classified in the same group as medulloblastomas as CNS WHO grade 4 embryonal tumours and are highly malignant [74] (Fig. 2). There is currently no international consensus for the standard treatment of ATRT, but it generally involves a multimodal approach of surgery, chemotherapy, and radiotherapy [74]. Due to this, prognosis is often poor with an average 5-year survival rate of 42%, thus there is a need for improved treatment options [58].

ATRT tumours have an especially low mutation rate compared to other brain cancers [5]. It was previously thought that *SMARCB1* was the exclusive recurrent mutation that characterised ATRT tumours, either through germline or somatic *SMARCB1* mutations, or deletions on chromosome 22q [75]. *SMARCB1* is a core subunit of the SWI/SNF complex and functions as a tumour suppressor gene [76]. Loss of *SMARCB1* expression leads to ATRTs without the side-effect of massive genomic instability that is observed with some tumour types [76]. Whilst *SMARCB1* remains the main genetic aberration that characterises ATRTs, it is now known that *SMARCA4* mutations occur in a rare number of ATRT cases where *SMARCB1* expression remains present [77–80]. Multiple studies have reported that ATRT patients who retained positive nuclear staining for *SMARCB1* in tumour cells lacked staining for *SMARCA4* [77–79]. *SMARCA4* mutations observed in ATRT are commonly homozygous and inactivating, which is characteristic of a tumour suppressor gene [5, 78].

Whilst the genetic background of ATRTs is relatively simple, the epigenetic profile of this tumour type is far more complex [81]. Three distinct molecular subgroups of ATRT have been identified that are defined by clinical features, patient demographic and tumour location, in combination with gene expression and DNA methylation signatures [33, 74, 82]. The subtypes are named according to the specific molecular pathways that are overexpressed; TYR, SHH, and MYC [33]. The TYR subgroup is characterised by overexpression of melanosomal genes and tumours predominantly occur in the infratentorial region, the SHH group has overexpression of the Sonic Hedgehog pathway and occur equally in infra- and supratentorial locations, and the MYC group overexpresses genes in the MYC and HOX cluster and are most commonly supratentorial tumours [33]. Overexpression of the SHH pathway is a defining feature of brain cancers such as ATRT and MB, described above. One study profiled 192 ATRT tumours and identified 3 tumours that showed retained *SMARCB1* expression, with all 3 of these tumours carrying a mutation in *SMARCA4* and clustering in the SHH-subgroup of ATRT [33]. This may indicate that there is an association between *SMARCA4* mutation and altered expression of the SHH pathway that is specific to this ATRT subgroup. Whilst this is a small proportion (*n* = 3) of total ATRT-SHH tumours (*n* = 65), *SMARCA4* mutation does occur at a much lower rate compared to *SMARCB1* in ATRT and its exclusivity to the SHH subgroup is notable. Comparatively, *SMARCA4* mutation is rarely seen in the SHH subgroup of MB and is a crucial regulator of underlying epigenetic networks of this tumour type which are required for tumourigenesis [30]. This is likely due to the fact that *SMARCA4* is reported to have a tumour suppressing role in ATRT, whereas it is known to be an oncogenic driver in SHH-group MB. Whilst the way *SMARCA4* and the SHH pathway are interacting is likely different between ATRT and SHH-MB, the mechanism could similarly be through epigenetic regulation with differences caused by the type of epigenetic marks that are modified.

## Clinical SMARCA4 mutations reported in brain cancer

As described above, *SMARCA4* mutations observed in brain cancer are largely dependent on the type and clinical subgroup of the tumour, displaying diverse genetic interactions and functional consequences. *SMARCA4* generally appears to play a tumour suppressor role in MB and ATRT as opposed to having the function of an oncogene in GBM. However, there are several exceptions to this. Patient mutation data from online databases cBioPortal (https://www.cbioportal.org/) and COSMIC (https://cancer.sanger.ac.uk/cosmic3d/) were reviewed to determine the most common *SMARCA4* genetic alterations specific to MB, ATRT, oligodendroglioma (low-grade glioma) and GBM (high-grade glioma).

**Fig. 3:**
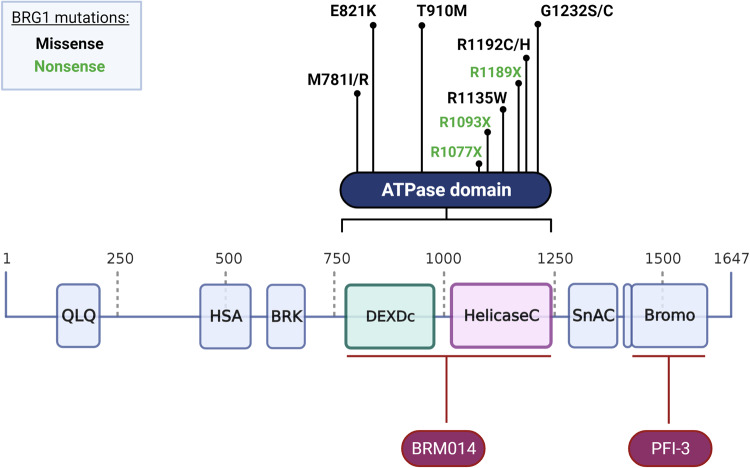
Positions of BRG1 ATPase domain mutations reported in brain cancer and the targets of BRG1 inhibitors. BRG1 is a 1647 amino acid protein encoded by the *SMARCA4* gene, with the ATPase domain spanning amino acids 750–1250. DEAD-like helicases superfamily domain (DEXDc) and helicase superfamily c-terminal domain (HelicaseC) provide the catalytic activity of the ATPase domain. BRM014 inhibits BRG1 via targeting the ATPase domain, whilst the inhibitor PFI-3 targets the bromodomain. Created with BioRender.com.

In SHH and Group 4 subgroups of MB, *SMARCA4* is very rarely mutated, with little to no *SMARCA4* patient mutations recorded. However, it is well documented that WNT and Group 3 MB tumours commonly experience loss-of-function missense *SMARCA4* mutations that occur in the ATPase domain. Recurrent missense mutations identified in MB tumours include M781I/R, E821K, T910M, R1135W and G1232S/C (Fig. 3, Table 1) [31, 36, 38, 48, 83–86]. These missense mutations are some of the most frequently reported *SMARCA4* mutations across all cancers [18, 20]. Inactivating missense mutations of *SMARCA4* in WNT and Group 3 subgroups of MB are usually heterozygous [31].

**Table 1:** A summary of *SMARCA4* ATPase domain mutations reported in clinical presentations of brain cancer by tumour type.

Tumour type	SMARCA4 mutation	Mutation type	Mutational consequence	% of SMARCA4 mutated samples	References
Medulloblastoma (MB)	M781I/R	Missense	Predicted oncogenic	5% (2/43)	Northcott et al. 2017 [84]
	E821K	Missense	Predicted oncogenic	5% (2/43)	Jones et al. 2012 Robinson et al. 2012 [31, 38]
	T910M	Missense	Predicted oncogenic loss-of-function	21% (9/43)	Parsons et al. 2011 Jones et al. 2012 Pugh et al. 2012 Robinson et al. 2012 Parsons et al. 2016 Northcott et al. 2017 [31, 36, 38, 48, 84, 85]
	R1135W	Missense	Predicted oncogenic	5% (2/43)	Jones et al. 2012 [38]
	G1232S/C	Missense	Predicted oncogenic loss-of-function	9% (4/43)	Parsons et al. 2011 Jones et al. 2012 Robinson et al. 2012 Wong et al. 2020 [31, 36, 38, 86]
Oligodendroglioma	M781I	Missense	Predicted oncogenic	8% (2/25)	Jonsson et al. 2019 [69]
	T910M	Missense	Predicted oncogenic loss-of-function	8% (2/25)	Zehir et al. 2017 Jonsson et al. 2019 [68, 69]
	R1192C/H	Missense	Predicted oncogenic	12% (3/25)	Aihara et al. 2017 Hoadley et al. 2018 Jonsson et al. 2019 [67, 69, 70]
	G1232S	Missense	Predicted oncogenic	8% (2/25)	Thomas et al. 2017 Jonsson et al. 2019 [69, 71]
Atypical teratoid/rhabdoid tumour (ATRT)	R1077X	Nonsense	Predicted oncogenic	25% (1/4)	Witkowski et al. 2013 [80]
	R1093X	Nonsense	Known oncogenic	25% (1/4)	Bookhout et al. 2018 [79]
	R1189X	Nonsense	Predicted severely truncated protein or nonsense-mediated decay	25% (1/4)	Schneppenheim et al. 2010 [78]

Patient tumour data was collated from cBioPortal and COSMIC online databases. Only recurrent mutations were included for MB and oligodendroglioma, total unique samples carrying a *SMARCA4* mutation were *n* = 43 and *n* = 25 respectively. Mutations for ATRT tumours were *n* = 1 from a total of 4 mutated samples.

*SMARCA4* is known to similarly play a tumour suppressor role in ATRT. In contrast to MB, *SMARCA4* mutations in ATRT are largely homozygous nonsense mutations; defined as point mutations that result in a premature stop sequence, usually resulting in an incomplete protein product [77–79]. *SMARCA4* mutations that have been reported in ATRT patients include nonsense mutations Q678X, and R1077X, R1093X and R1189X which occur in the ATPase domain (Fig. 3, Table 1) [77–80]. The majority of these mutations have only been reported in a single patient as *SMARCA4*-deficient tumours make up a small subset of ATRT cases, yet they share characteristics such as location and mutation type. These ATRT nonsense mutations are suggested to produce a truncated BRG1 protein that is removed via nonsense-mediated decay [78, 79]. Loss of *SMARCA4* is sometimes the sole oncogenic event in ATRT and causes complete loss-of-function, compared to MB where *SMARCA4* is still expressed but mutation instead affects functionality of the protein [37]. Thus, despite *SMARCA4* functioning as a tumour suppressor in both of these brain cancers, the mechanism via which *SMARCA4* mutation is involved in tumourigenesis may be unique and occur at different stages of tumour progression.

The frequency of *SMARCA4* mutations in gliomas appear to differ between low and high-grade tumours. Far more recurrent missense mutations have been reported in low-grade oligodendroglioma compared to GBM, which is a highly aggressive tumour. Recurrent oligodendroglioma patient mutations include M781I, T910M, R1192C/H and G1232S, again all located in the ATPase domain of *SMARCA4* and bearing resemblance to mutations reported in MB (Fig. 3, Table 1) [67–71]. In GBM, there were no recurrent ATPase domain point mutations identified. However, multiple non-recurrent *SMARCA4* ATPase missense mutations were reported which contradicts existing literature that suggests *SMARCA4* is rarely altered at the genetic level in GBM [64]. Interestingly, *SMARCA4* amplification and overexpression were reported at a greater frequency in GBM compared to other brain cancer types. Increased expression of BRG1 is a characteristic feature of GBM [20, 64]. Therefore, it is more likely that amplification of *SMARCA4* is causing altered expression rather than missense mutations which appear to occur more sporadically in GBM than in other brain cancers. The differences observed in the frequency and type of *SMARCA4* genetic alterations between oligodendroglioma and GBM are in line with evidence that suggests *SMARCA4* mutation is associated with an increased risk of oligodendroglioma [32], compared to GBM where BRG1 is thought to play an oncogenic role and maintain stemness of GICs [59, 61].

## Functional studies of SMARCA4 mutations in vitro and in vivo

*SMARCA4* mutations in brain cancer are well documented, however, less is known about how these mutations functionally alter BRG1 function on a molecular level to influence tumourigenesis. Whilst numerous studies have investigated the effects of BRG1 overexpression and knockdown in the context of brain cancer [30, 61, 63, 64], fewer have explored the direct mechanistic consequences that specific point mutations recurrently observed in brain cancer patients have on normal BRG1 function. Heterozygous *SMARCA4* missense mutations have previously been modeled in yeast to assess positional effects on chromatin remodeling capacity [20]. Overall, it was shown that a greater proportion of DNA accessibility losses were detected in *SMARCA4* mutants compared to accessibility gains, consistent with BRG1 most commonly being referred to as a chromatin opener [20]. Specific positional effects were also observed, with mutations in the DNA binding domain preventing BRG1 binding to nucleosomes, whereas ATPase domain mutants prevented BRG1 release from chromatin [20]. An additional consequence of *SMARCA4* ATPase domain mutation previously reported is the increase in genome wide PRC1 binding; a known transcriptional repressor [87]. In relation to cancer, it has been demonstrated in non-small cell lung cancer cells that *SMARCA4* missense mutations in the ATPase domain similarly reduced nucleosome remodeling activity compared to the wild-type cells [18]. Whilst the mechanisms of a subset of *SMARCA4* mutations have been explored, the functional consequence of many of the mutations listed in Table 1 are still yet to be determined. This will be a crucial step in the development of therapies to target these tumours.

Functional studies of *SMARCA4* mutation in brain cancer models have been very limited. A 2010 study recombinantly overexpressed a *SMARAC4* mutation in non-brain cancer cell lines that had been derived from an ATRT patient. It was observed that the R1189X mutation resulted in expression of an aberrant truncated protein which was clearly defined from wild-type BRG1 [78]. This suggests that although the mutant allele of *SMARCA4* can be successfully translated into messenger RNA, nonsense-mediated decay of the truncated protein may cause complete loss of BRG1 expression in ATRT tumour cells that carry this mutation [78]. It is likely that other similar ATRT *SMARCA4* nonsense mutations affecting an arginine residue in the ATPase domain such as R1077X and R1093X also produce a truncated protein [77, 79].

Whilst missense *SMARCA4* mutations observed in MB and oligodendroglioma have not been investigated in their native setting, they have been studied in human embryonic kidney cells. Missense mutations E821K, T910M, R1192C and G1232S (listed in Table 1) were included in a panel of *SMARCA4* mutants and displayed inhibited remodeling capacity compared to wild-type cells [18]. The significant effect of these mutations is likely due to their position in the highly conserved ATPase domain and the severity of amino acid changes [18]. E821K and R1192C mutations were predicted to change the charge of the protein residue at this site and G1232S was expected to modify polarity, hence altering the physiochemical properties of the BRG1 protein and inhibiting normal activity [18]. The T910M *SMARCA4* mutation has been further investigated in a small cell carcinoma of the ovary hypercalcemic type (SCCOHT) cell line [88]. In the ATPase deficient SCCOHT cell line BIN-67, which lacks both BRG1 and BRM expression, introduction of T910M mutant *SMARCA4* showed similar protein expression to when wild-type *SMARCA4* was expressed [88]. However, the T910M mutant exhibited partial loss of catalytic activity and a reduced affinity to chromatin of SWI/SNF complexes [88]. Additionally, the authors suggested that BRG1 is required for functional specification and correct genome localisation of BAF and PBAF complexes, with only reintroduction of wild-type *SMARCA4*, not the T910 mutant, shown to restore DNA accessibility and paralog-specific localisation of SWI/SNF complexes in the genome [88]. Whilst the SCCOHT cell line is a unique example that does not entirely reflect brain cancer, in brain cancer the *SMARCA4* T910M mutation is primarily heterozygous and occurs in the presence of BRM expression, findings from this study reveal key mechanistic insights. It appears *SMARCA4* mutation has the ability to affect SWI/SNF complex activity at a direct molecular binding level, but also at a larger complex level where configuration and balanced expression of BAF, PBAF and ncBAF complexes may be altered. The three paralogs of the human SWI/SNF complex uniquely localise to different chromatin sites [89]. BAF complexes preferentially target active enhancers, PBAF complexes target active promoters and gene bodies, and ncBAF complexes localise at CTCF sites and promoters [89]. Therefore, lack of complex identity due to *SMARAC4* mutation is likely to cause incorrect targeting and dysregulation of transcriptional programs. Although *SMARCA4* mutations were assessed in non-brain cancer cell lines, these studies provide hypothesis-generating results that suggest missense mutations may disrupt BRG1 function in brain cancer in a similar way and will inform brain cancer research in the future.

## Therapeutic strategies to target SMARCA4 in brain cancer

Due to the mutual exclusivity of *SMARCA4* and *SMARCA2* as catalytic subunits of the SWI/SNF complex, a common approach to targeted treatment of *SMARCA4*-mutant cancers is synthetic lethality. This approach harnesses the simultaneous mutation of two genes to induce cell death that would otherwise not happen if one of the mutations occurred alone [90]. In these cancers, *SMARCA4* has a tumour-supressing quality and mutation is thought to contribute to cancer development. It has previously been found that BRG1 inactivation leads to increased incorporation of BRM into the SWI/SNF complex [91]. Therefore, *SMARCA2* is an attractive target to inhibit in *SMARCA4*-mutant cancers, utilising the enhanced reliance of tumour cells on BRM to completely prevent activity of the SWI/SNF chromatin remodeling complex and inhibit cancer cell growth [92]. This method has been successfully demonstrated to supress the growth of non-small cell lung cancer lacking *SMARCA4* in vitro and in vivo through xenograft mouse models [93, 94]. A 2018 study was the first to discover orally active inhibitors of the *SMARCA2* ATPase domain and showed that they had anti-proliferative effects in a *SMARCA4*-mutant lung cancer xenograft mouse model [56]. It has since been demonstrated that BRG1 catalytic activity is also inhibited by the same inhibitor in mouse embryonic stem cells where BRM is very weakly expressed and thus BRG1 constitutes the major SWI/SNF ATPase [95]. Previous to this, no small molecules had been reported to modulate SWI/SNF complex activity via ATPase domain inhibition. The small molecules were described to modulate ATPase activity via allosteric inhibition, causing a change in shape of the protein and thus altered function [56]. The inhibitor, known as BRM014, is a dual inhibitor of both BRM and BRG1 [56]. In the case of *SMARCA4*-deficient cancers, the principle of synthetic lethality preferentially targets these cancer cells. However, the inability to separate the inhibitory activity against BRM and BRG1 could have unwanted effects and potential side-effects in normal cells must be carefully reviewed in any clinical trial. Development of these small molecule inhibitors is a significant step in *SMARCA4/SMARCA2* targeted therapies that with refinement and further validation could progress to pre-clinical trials. Whilst *SMARCA2* inhibition has been demonstrated as a successful therapeutic strategy in *SMARCA4*-deficient cancer cell lines, its viability is yet to be confirmed in brain cancer cell lines where *SMARCA4* is frequently inactivated.

In brain cancers where BRG1 has a crucial role in tumour cell progression such as SHH-group MB and GBM, synthetic lethality is not a feasible strategy as BRG1 retains its function in the wild-type form. Either BRG1 overexpression in GBM or aberrant activity in SHH-MB appears to drive key cancer-promoting pathways. The fundamental molecular mechanisms underlying oncogenic changes driven by BRG1 overexpression are yet to be extensively documented. However, we speculate that the greatest effects on cell transformation may come from disrupting the balance of SWI/SNF complex formation which can alter complex abundance, chromatin targeting and ultimately gene expression. In a similar way in which BRM incorporation is known to compensate for BRG1 loss in BRG1-mutant cancers [91], BRG1 overexpression may reduce the number of BRM containing SWI/SNF complexes and caused preferential ATPase recruitment. Whilst large overlap between the genomic sites that BRM and BRG1 occupy has been reported, there are factors and loci that associate differently with the two ATPases that may be affected by BRG1 inhibition [96]. Alternatively, BRG1 overexpression may increase the total number of SWI/SNF complexes with potential for excess complexes to target novel sites in the genome. Changes may also exist at a SWI/SNF subtype level with BRG1 recruiting to BAF, PBAF and ncBAF complexes at different frequencies, potentially altering specificity and activity of these complexes. To prevent oncogenic activity of BRG1, sole BRG1 inhibition may be another avenue of therapeutic intervention, yet this has proved to be more challenging. A previous study has demonstrated that inhibition of BRG1 function via the PFI-3 inhibitor, targeting the bromodomain (Fig. 3), increased the radiosensitivity of colon cancer cells in vitro and in a xenograft mouse model resulting in increased tumour cell death following irradiation [97]. However, the focus of this study was the post-irradiation response of tumour cells and therefore baseline effects on cell proliferation in the absence of additional therapeutic intervention was not investigated. Recently it has been suggested that PFI-3 is in fact most effective when used in combination with other therapies by sensitising cells to DNA damage [98]. Whilst on its own PFI-3 had little effect, when combined with chemotherapeutic agents such as doxorubicin, PFI-3 was able to successfully increase the sensitivity of several human cancer cell lines to chemotherapy-induced DNA damage [98]. BRG1 phosphorylation is a critical event that occurs rapidly after DNA damage and allows BRG1 to bind γ-H2AX sites and form repair foci, thus inhibition of BRG1 impedes this response [99]. Whilst Kwon et al. successfully supressed BRG1 activity via the bromodomain, it has been suggested that the ATPase domain may be a better target as catalytic BRG1 ATPase domain activity is essential for SWI/SNF function and tumour cell growth [100]. The previously mentioned novel small molecule inhibitor, BRM014, targets the ATPase domain of BRG1 (Fig. 3), and its activity has recently been trialled in multiple human cell lines showing promising SWI/SNF inhibiting effects [101, 102]. However, its activity is yet to be tested in brain cancer cell lines. Pharmacological inhibition of the ATPase domain could potentially produce greater tumour cell inhibiting effects compared to targeting the bromodomain. Understanding the direct oncogenic mechanisms by which BRG1 is contributing to tumour development, whether that be via dysregulation of specific transcriptional programs, abnormal SWI/SNF complex recruitment and targeting, or an impaired DNA damage response, will guide treatment development in the most effective way.

There is a degree of difficulty that exists in inhibiting BRG1 due to barriers in the molecular structure. An alternative therapeutic strategy to small molecule inhibitors is CRISPR/Cas9 gene therapy [103, 104]. As well as achieving highly targeted BRG1 inhibition, this novel strategy could potentially enable the sole inhibition of BRG1 separately to BRM, which has proved difficult to achieve through chemical inhibition. CRISPR knock-out has been successfully demonstrated for tumour-suppressor genes in brain tumour modelling [105], with potential for the same principles to be applied to a therapeutic approach for BRG1 inhibition in brain cancers where BRG1 exhibits oncogenic functions. A recent 2022 study developed a non-invasive gene therapy delivery method for brain cancers, creating nanocapsules which effectively and safely delivered the CRISPR/Cas9 complex across the blood-brain barrier [103]. Using both the GBM cell line U87MG and U87MG-Luc GBM mice, the GBM associated gene *PLK1* was successfully edited following nanocapsule delivery of a gene-specific guide RNA and mice treated with nanocapsules showed significantly inhibited tumour growth [103]. This provides valuable in vivo evidence of successful CRISPR/Cas9 gene editing with negligible off-target effects in GBM that could be a feasible strategy in other brain cancers for the targeting of BRG1.

The significant association of BRG1 with brain cancers makes it an attractive therapeutic target. However, as with any therapy, there is potential for side-effects in normal tissues to occur. As described in this review, the role BRG1 plays in tumourigenesis, and the genetic alterations observed are highly dependent on cancer type. Therefore, therapies in turn are likely to be specialised to target a subset of mutations. The ultimate aim of inhibiting BRG1 is to prevent oncogenic action or cause cancer cell death, yet it also has the potential to drive wide-spread transcriptional changes. Residual subunits may maintain SWI/SNF function to some degree, but with decreased genome binding specificity [88]. This could involve altered expression of a number of other pathways that are regulated by SWI/SNF complex activity, making specific side-effects hard to predict. Finding ways to preferentially target cancer cells, for example through increased BRM reliance, increased proliferation rates or highly specific delivery methods will be a valuable strategy in reducing off-target effects in the surrounding normal tissue. A major aspect of pre-clinical trials will be monitoring for these unwanted side-effects, as well as optimising dosage to effectively inhibit aberrant BRG1 activity whilst preserving normal SWI/SNF function in non-cancerous cells.

In addition to its role in tumourigenesis, BRG1 has been shown to have a time-specific function in cerebral precursor cell development [52]. This raises concerns about BRG1 inhibition having potentially detrimental effects in the younger brain cancer patient group. BRG1 is ubiquitously expressed, and the literature suggests that BRG1 involvement in tumourigenesis may be preceded by a crucial role in cerebral development [54]. As many brain cancer patients are infants and young children, the onset of tumours often coincides with the critical developmental period of the cerebellum which continues until 2 years-of-age [106]. The effects of using direct BRG1 inhibition as a therapeutic strategy in vivo are slowly emerging but greater knowledge is required to guide pre-clinical trials. A major area of focus should be in determining the specificities of when BRG1 action initiates tumour development in brain cancers such as MB, establishing the safest window to receive treatment whilst also being highly effective at preventing early tumour events. This will assist in the development of time-dependent and selective BRG1 inhibitors that can be viable for a wide-range of patients.

## Conclusions and future directions

Overall, *SMARCA4*/BRG1 function and the genetic pathways it regulates are crucial to the underlying molecular mechanisms that are involved in brain cancer. BRG1 plays a tumour supressing role in ATRT and WNT and Group 3 MB, with loss-of-function mutations driving tumour initiation and development. In contrast, BRG1 activity in SHH-MB and GBM is observed to promote the cancer phenotype. *SMARCA4* mutations have varying effects that are largely dependent on tumour and mutation type. Heterozygous missense *SMARCA4* mutations in the ATPase domain are most commonly observed in WNT and Group 3 MB, whereas *SMARCA4* is rarely mutated in SHH and Group 4. Homozygous nonsense *SMARCA4* mutations also in the ATPase domain are most frequently observed in ATRT tumours which retain *SMARCB1* expression. BRG1 overexpression is commonly observed in GBM, however *SMARCA4* mutation is rare and thus it is likely that altered expression is caused via other mechanisms that may be epigenetic. Due to the heterogeneous role of BRG1 in brain cancer, it is likely that therapies will have to be developed to target specific mutations in a subset of tumour types. This will require further understanding of the BRG1-regulated pathways that underpin tumour development and progression. Strategies such as synthetic lethality and the development of small-molecule inhibitors show encouraging signs that BRG1 can be successfully targeted through therapeutic intervention, and CRISPR/Cas9 gene editing is emerging as a novel strategy; all with the potential to act as adjuvant therapy to improve current standard treatments for brain cancer. Chromatin remodeling is known to be a major regulator of cancer gene expression programs, therefore knowledge of BRG1 involvement and targeted strategies developed in brain cancer are likely to be widely applicable to a variety of other cancer types where BRG1 has a clear role in tumourigenesis such as lung, ovarian and prostate cancer.

## References

[1] Dawson MA, Kouzarides T (2012). Cancer epigenetics: from mechanism to therapy. Cell.

[2] Kagohara LT, Stein-O’Brien GL, Kelley D, Flam E, Wick HC, Danilova LV (2018). Epigenetic regulation of gene expression in cancer: techniques, resources and analysis. Brief Funct Genom.

[3] Baylin SB, Jones PA (2016). Epigenetic determinants of cancer. Cold Spring Harb Perspect Biol.

[4] Roy DM, Walsh LA, Chan TA (2014). Driver mutations of cancer epigenomes. Protein Cell.

[5] Lee RS, Stewart C, Carter SL, Ambrogio L, Cibulskis K, Sougnez C (2012). A remarkably simple genome underlies highly malignant pediatric rhabdoid cancers. J Clin Invest.

[6] Alfert A, Moreno N, Kerl K (2019). The BAF complex in development and disease. Epigenetics Chromatin.

[7] Kumar R, Li DQ, Müller S, Knapp S (2016). Epigenomic regulation of oncogenesis by chromatin remodeling. Oncogene.

[8] Giles KA, Taberlay PC (2019). Mutations in chromatin remodelling factors. Encyclopedia of Cancer [Internet]..

[9] Kadoch C, Hargreaves DC, Hodges C, Elias L, Ho L, Ranish J (2013). Proteomic and bioinformatic analysis of mammalian SWI/SNF complexes identifies extensive roles in human malignancy. Nat Genet.

[10] Mashtalir N, D'Avino AR, Michel BC, Luo J, Pan J, Otto JE (2018). Modular organization and assembly of SWI/SNF family chromatin remodeling complexes. Cell.

[11] Alpsoy A, Dykhuizen EC (2018). Glioma tumor suppressor candidate region gene 1 (GLTSCR1) and its paralog GLTSCR1-like form SWI/SNF chromatin remodeling subcomplexes. J Biol Chem.

[12] Mashtalir N, Dao HT, Sankar A, Liu H, Corin AJ, Bagert JD (2021). Chromatin landscape signals differentially dictate the activities of mSWI/SNF family complexes. Science.

[13] Qi W, Wang R, Chen H, Wang X, Xiao T, Boldogh I (2015). BRG1 promotes the repair of DNA double-strand breaks by facilitating the replacement of RPA with RAD51. J Cell Sci.

[14] Qi W, Chen H, Lu C, Bu Q, Wang X, Han L (2018). BRG1 promotes chromatin remodeling around DNA damage sites. Anim Cells Syst (Seoul).

[15] Hays E, Nettleton E, Carter C, Morales M, Vo L, Passo M (2020). The SWI/SNF ATPase BRG1 stimulates DNA end resection and homologous recombination by reducing nucleosome density at DNA double strand breaks and by promoting the recruitment of the CtIP nuclease. Cell Cycle.

[16] Kurashima K, Kashiwagi H, Shimomura I, Suzuki A, Takeshita F, Mazevet M (2020). SMARCA4 deficiency-associated heterochromatin induces intrinsic DNA replication stress and susceptibility to ATR inhibition in lung adenocarcinoma. NAR Cancer.

[17] Gupta M, Concepcion CP, Fahey CG, Keshishian H, Bhutkar A, Brainson CF (2020). BRG1 loss predisposes lung cancers to replicative stress and ATR dependency. Cancer Res.

[18] Fernando TM, Piskol R, Bainer R, Sokol ES, Trabucco SE, Zhang Q (2020). Functional characterization of SMARCA4 variants identified by targeted exome-sequencing of 131,668 cancer patients. Nat Commun.

[19] Peng L, Li J, Wu J, Xu B, Wang Z, Giamas G (2021). A pan-cancer analysis of SMARCA4 alterations in human cancers. Front Immunol.

[20] Hodges HC, Stanton BZ, Cermakova K, Chang CY, Miller EL, Kirkland JG (2018). Dominant-negative SMARCA4 mutants alter the accessibility landscape of tissue-unrestricted enhancers. Nat Struct Mol Biol.

[21] Wu Q, Lian JB, Stein JL, Stein GS, Nickerson JA, Imbalzano AN (2017). The BRG1 ATPase of human SWI/SNF chromatin remodeling enzymes as a driver of cancer. Epigenomics.

[22] Fillmore CM, Xu C, Desai PT, Berry JM, Rowbotham SP, Lin YJ (2015). EZH2 inhibition sensitizes BRG1 and EGFR mutant lung tumours to TopoII inhibitors. Nature.

[23] Witkowski L, Carrot-Zhang J, Albrecht S, Fahiminiya S, Hamel N, Tomiak E (2014). Germline and somatic SMARCA4 mutations characterize small cell carcinoma of the ovary, hypercalcemic type. Nat Genet.

[24] Hodis E, Watson IR, Kryukov GV, Arold ST, Imielinski M, Theurillat JP (2012). A landscape of driver mutations in melanoma. Cell.

[25] Love C, Sun Z, Jima D, Li G, Zhang J, Miles R (2012). The genetic landscape of mutations in Burkitt lymphoma. Nat Genet.

[26] Shi J, Whyte WA, Zepeda-Mendoza CJ, Milazzo JP, Shen C, Roe JS (2013). Role of SWI/SNF in acute leukemia maintenance and enhancer-mediated Myc regulation. Genes Dev.

[27] Giles KA, Gould CM, Achinger-Kawecka J, Page SG, Kafer GR, Rogers S (2021). BRG1 knockdown inhibits proliferation through multiple cellular pathways in prostate cancer. Clin Epigenet.

[28] Sobczak M, Pietrzak J, Ploszaj T, Robaszkiewicz A (2020). BRG1 activates proliferation and transcription of cell cycle-dependent genes in breast cancer cells. Cancers (Basel).

[29] Wu Q, Madany P, Akech J, Dobson JR, Douthwright S, Browne G (2015). The SWI/SNF ATPases are required for triple negative breast cancer cell proliferation. J Cell Physiol.

[30] Shi X, Wang Q, Gu J, Xuan Z, Wu JI (2016). SMARCA4/Brg1 coordinates genetic and epigenetic networks underlying Shh-type medulloblastoma development. Oncogene.

[31] Robinson G, Parker M, Kranenburg TA, Lu C, Chen X, Ding L (2012). Novel mutations target distinct subgroups of medulloblastoma. Nature.

[32] Amankwah EK, Thompson RC, Nabors LB, Olson JJ, Browning JE, Madden MH (2013). SWI/SNF gene variants and glioma risk and outcome. Cancer Epidemiol.

[33] Johann PD, Erkek S, Zapatka M, Kerl K, Buchhalter I, Hovestadt V (2016). Atypical teratoid/rhabdoid tumors are comprised of three epigenetic subgroups with distinct enhancer landscapes. Cancer Cell.

[34] Patel AP, Fisher JL, Nichols E, Abd-Allah F, Abdela J, Abdelalim A (2019). Global, regional, and national burden of brain and other CNS cancer, 1990–2016: a systematic analysis for the Global Burden of Disease Study 2016. Lancet Neurol.

[35] McNeill KA (2016). Epidemiology of brain tumors. Neurol Clin.

[36] Parsons DW, Li M, Zhang X, Jones S, Leary RJ, Lin JC (2011). The genetic landscape of the childhood cancer medulloblastoma. Science.

[37] Johann PD (2020). Invited review: dysregulation of chromatin remodellers in paediatric brain tumours - SMARCB1 and beyond. Neuropathol Appl Neurobiol.

[38] Jones DT, Jager N, Kool M, Zichner T, Hutter B, Sultan M (2012). Dissecting the genomic complexity underlying medulloblastoma. Nature.

[39] Millard NE, De Braganca KC (2016). Medulloblastoma. J Child Neurol.

[40] Wells EM, Packer RJ. Pediatric brain tumors. Continuum (Minneap Minn). 2015;21(2 Neuro-oncology):373–96. 10.1212/01.CON.0000464176.96311.d1 25837902

[41] Louis DN, Perry A, Wesseling P, Brat DJ, Cree IA, Figarella-Branger D (2021). The 2021 WHO classification of tumors of the central nervous system: a summary. Neuro Oncol.

[42] Northcott PA, Hielscher T, Dubuc A, Mack S, Shih D, Remke M (2011). Pediatric and adult sonic hedgehog medulloblastomas are clinically and molecularly distinct. Acta Neuropathol.

[43] Northcott PA, Robinson GW, Kratz CP, Mabbott DJ, Pomeroy SL, Clifford SC (2019). Medulloblastoma. Nat Rev Dis Prim.

[44] Louis DN, Perry A, Reifenberger G, von Deimling A, Figarella-Branger D, Cavenee WK (2016). The 2016 World Health Organization classification of tumors of the central nervous system: a summary. Acta Neuropathol.

[45] Cavalli FMG, Remke M, Rampasek L, Peacock J, Shih DJH, Luu B (2017). Intertumoral heterogeneity within medulloblastoma subgroups. Cancer Cell.

[46] Hovestadt V, Ayrault O, Swartling FJ, Robinson GW, Pfister SM, Northcott PA (2020). Medulloblastomics revisited: biological and clinical insights from thousands of patients. Nat Rev Cancer.

[47] Northcott PA, Korshunov A, Witt H, Hielscher T, Eberhart CG, Mack S (2011). Medulloblastoma comprises four distinct molecular variants. J Clin Oncol.

[48] Pugh TJ, Weeraratne SD, Archer TC, Pomeranz Krummel DA, Auclair D, Bochicchio J (2012). Medulloblastoma exome sequencing uncovers subtype-specific somatic mutations. Nature.

[49] Yi J, Wu J (2018). Epigenetic regulation in medulloblastoma. Mol Cell Neurosci.

[50] Archer TC, Mahoney EL, Pomeroy SL (2017). Medulloblastoma: molecular classification-based personal therapeutics. Neurotherapeutics.

[51] Shi X, Zhang Z, Wang Q, Wu J (2015). Function of Brg1 chromatin remodeling factor in sonic hedgehod-dependent medulloblastoma initiation and maintenance. Mol Cell Biol.

[52] Moreno N, Schmidt C, Ahlfeld J, Poschl J, Dittmar S, Pfister SM (2014). Loss of Smarc proteins impairs cerebellar development. J Neurosci.

[53] Sun G, Rong D, Li Z, Sun G, Wu F, Li X (2021). Role of small molecule targeted compounds in cancer: progress, opportunities, and challenges. Front Cell Dev Biol.

[54] Holdhof D, On JH, Schoof M, Gobel C, Schuller U (2021). Simultaneous Brg1 knockout and MYCN overexpression in cerebellar granule neuron precursors is insufficient to drive tumor formation but temporarily enhances their proliferation and delays their migration. Cerebellum.

[55] Holdhof D, Schoof M, Hellwig M, Holdhof NH, Niesen J, Schuller U (2020). hGFAP-positive stem cells depend on Brg1 for proper formation of cerebral and cerebellar structures. Cereb Cortex.

[56] Papillon JPN, Nakajima K, Adair CD, Hempel J, Jouk AO, Karki RG (2018). Discovery of orally active inhibitors of brahma homolog (BRM)/SMARCA2 ATPase activity for the treatment of brahma related gene 1 (BRG1)/SMARCA4-mutant cancers. J Med Chem.

[57] Chen R, Smith-Cohn M, Cohen AL, Colman H (2017). Glioma subclassifications and their clinical significance. Neurotherapeutics.

[58] Ostrom QT, Cioffi G, Gittleman H, Patil N, Waite K, Kruchko C (2019). CBTRUS statistical report: primary brain and other central nervous system tumors diagnosed in the United States in 2012-6. Neuro Oncol.

[59] Hiramatsu H, Kobayashi K, Kobayashi K, Haraguchi T, Ino Y, Todo T (2017). The role of the SWI/SNF chromatin remodeling complex in maintaining the stemness of glioma initiating cells. Sci Rep.

[60] Olar A, Aldape KD (2014). Using the molecular classification of glioblastoma to inform personalized treatment. J Pathol.

[61] Ganguly D, Sims M, Cai C, Fan M, Pfeffer LM (2018). Chromatin remodeling factor BRG1 regulates stemness and chemosensitivity of glioma initiating cells. Stem Cells.

[62] Paugh BS, Qu C, Jones C, Liu Z, Adamowicz-Brice M, Zhang J (2010). Integrated molecular genetic profiling of pediatric high-grade gliomas reveals key differences with the adult disease. J Clin Oncol.

[63] Bai J, Mei P-J, Liu H, Li C, Li W, Wu Y-P (2012). BRG1 expression is increased in human glioma and controls glioma cell proliferation, migration and invasion in vitro. J Cancer Res Clin Oncol.

[64] Wang Y, Yang CH, Schultz AP, Sims MM, Miller DD, Pfeffer LM (2021). Brahma‐Related Gene‐1 (BRG1) promotes the malignant phenotype of glioblastoma cells. J Cell Mol Med.

[65] Zou S, Tong Q, Liu B, Huang W, Tian Y, Fu X (2020). Targeting STAT3 in cancer immunotherapy. Mol Cancer.

[66] Campos B, Olsen LR, Urup T, Poulsen HS (2016). A comprehensive profile of recurrent glioblastoma. Oncogene.

[67] Aihara K, Mukasa A, Nagae G, Nomura M, Yamamoto S, Ueda H (2017). Genetic and epigenetic stability of oligodendrogliomas at recurrence. Acta Neuropathol Commun.

[68] Zehir A, Benayed R, Shah RH, Syed A, Middha S, Kim HR (2017). Mutational landscape of metastatic cancer revealed from prospective clinical sequencing of 10,000 patients. Nat Med.

[69] Jonsson P, Lin AL, Young RJ, DiStefano NM, Hyman DM, Li BT (2019). Genomic correlates of disease progression and treatment response in prospectively characterized gliomas. Clin Cancer Res.

[70] Hoadley KA, Yau C, Hinoue T, Wolf DM, Lazar AJ, Drill E (2018). Cell-of-origin patterns dominate the molecular classification of 10,000 tumors from 33 types of cancer. Cell.

[71] Thomas AA, Abrey LE, Terziev R, Raizer J, Martinez NL, Forsyth P (2017). Multicenter phase II study of temozolomide and myeloablative chemotherapy with autologous stem cell transplant for newly diagnosed anaplastic oligodendroglioma. Neuro Oncol.

[72] Mattick JS (2006). Makunin IV. Non-coding RNA. Hum Mol Genet.

[73] Anna A, Monika G (2018). Splicing mutations in human genetic disorders: examples, detection, and confirmation. J Appl Genet.

[74] Ho B, Johann PD, Grabovska Y, De Dieu Andrianteranagna MJ, Yao F, Fruhwald M (2020). Molecular subgrouping of atypical teratoid/rhabdoid tumors-a reinvestigation and current consensus. Neuro Oncol.

[75] Versteege I, Sevenet N, Lange J, Rousseau-Merck MF, Ambros P, Handgretinger R (1998). Truncating mutations of hSNF5/INI1 in aggressive paediatric cancer. Nature.

[76] McKenna ES, Sansam CG, Cho YJ, Greulich H, Evans JA, Thom CS (2008). Loss of the epigenetic tumor suppressor SNF5 leads to cancer without genomic instability. Mol Cell Biol.

[77] Hasselblatt M, Gesk S, Oyen F, Rossi S, Viscardi E, Giangaspero F (2011). Nonsense mutation and inactivation of SMARCA4 (BRG1) in an atypical teratoid/rhabdoid tumor showing retained SMARCB1 (INI1) expression. Am J Surg Pathol.

[78] Schneppenheim R, Fruhwald MC, Gesk S, Hasselblatt M, Jeibmann A, Kordes U (2010). Germline nonsense mutation and somatic inactivation of SMARCA4/BRG1 in a family with rhabdoid tumor predisposition syndrome. Am J Hum Genet.

[79] Bookhout C, Bouldin TW, Ellison DW (2018). Atypical teratoid/rhabdoid tumor with retained INI1 (SMARCB1) expression and loss of BRG1 (SMARCA4). Neuropathology.

[80] Witkowski L, Lalonde E, Zhang J, Albrecht S, Hamel N, Cavallone L (2013). Familial rhabdoid tumour 'avant la lettre'–from pathology review to exome sequencing and back again. J Pathol.

[81] Nesvick CL, Lafay-Cousin L, Raghunathan A, Bouffet E, Huang AA, Daniels DJ (2020). Atypical teratoid rhabdoid tumor: molecular insights and translation to novel therapeutics. J Neurooncol.

[82] Torchia J, Picard D, Lafay-Cousin L, Hawkins CE, Kim S-K, Letourneau L (2015). Molecular subgroups of atypical teratoid rhabdoid tumours in children: an integrated genomic and clinicopathological analysis. Lancet Oncol.

[83] Huether R, Dong L, Chen X, Wu G, Parker M, Wei L (2014). The landscape of somatic mutations in epigenetic regulators across 1000 paediatric cancer genomes. Nat Commun.

[84] Northcott PA, Buchhalter I, Morrissy AS, Hovestadt V, Weischenfeldt J, Ehrenberger T (2017). The whole-genome landscape of medulloblastoma subtypes. Nature.

[85] Parsons DW, Roy A, Yang Y, Wang T, Scollon S, Bergstrom K (2016). Diagnostic yield of clinical tumor and germline whole-exome sequencing for children with solid tumors. JAMA Oncol.

[86] Wong M, Mayoh C, Lau LMS, Khuong-Quang DA, Pinese M, Kumar A (2020). Whole genome, transcriptome and methylome profiling enhances actionable target discovery in high-risk pediatric cancer. Nat Med.

[87] Stanton BZ, Hodges C, Calarco JP, Braun SM, Ku WL, Kadoch C (2017). Smarca4 ATPase mutations disrupt direct eviction of PRC1 from chromatin. Nat Genet.

[88] Pan J, McKenzie ZM, D'Avino AR, Mashtalir N, Lareau CA, St Pierre R (2019). The ATPase module of mammalian SWI/SNF family complexes mediates subcomplex identity and catalytic activity-independent genomic targeting. Nat Genet.

[89] Michel BC, D'Avino AR, Cassel SH, Mashtalir N, McKenzie ZM, McBride MJ (2018). A non-canonical SWI/SNF complex is a synthetic lethal target in cancers driven by BAF complex perturbation. Nat Cell Biol.

[90] O'Neil NJ, Bailey ML, Hieter P (2017). Synthetic lethality and cancer. Nat Rev Genet.

[91] Wilson BG, Helming KC, Wang X, Kim Y, Vazquez F, Jagani Z (2014). Residual complexes containing SMARCA2 (BRM) underlie the oncogenic drive of SMARCA4 (BRG1) mutation. Mol Cell Biol.

[92] Hoffman GR, Rahal R, Buxton F, Xiang K, McAllister G, Frias E (2014). Functional epigenetics approach identifies BRM/SMARCA2 as a critical synthetic lethal target in BRG1-deficient cancers. Proc Natl Acad Sci USA.

[93] Oike T, Ogiwara H, Nakano T, Yokota J, Kohno T (2013). Inactivating mutations in SWI/SNF chromatin remodeling genes in human cancer. Jpn J Clin Oncol.

[94] Rago F, DiMare MT, Elliott G, Ruddy DA, Sovath S, Kerr G (2019). Degron mediated BRM/SMARCA2 depletion uncovers novel combination partners for treatment of BRG1/SMARCA4-mutant cancers. Biochem Biophys Res Commun.

[95] Iurlaro M, Stadler MB, Masoni F, Jagani Z, Galli GG, Schubeler D (2021). Mammalian SWI/SNF continuously restores local accessibility to chromatin. Nat Genet.

[96] Raab JR, Runge JS, Spear CC, Magnuson T (2017). Co-regulation of transcription by BRG1 and BRM, two mutually exclusive SWI/SNF ATPase subunits. Epigenetics Chromatin.

[97] Kwon SJ, Lee SK, Na J, Lee SA, Lee HS, Park JH (2015). Targeting BRG1 chromatin remodeler via its bromodomain for enhanced tumor cell radiosensitivity in vitro and in vivo. Mol Cancer Ther.

[98] Lee D, Lee DY, Hwang YS, Seo HR, Lee SA, Kwon J (2021). The bromodomain inhibitor PFI-3 sensitizes cancer cells to DNA damage by targeting SWI/SNF. Mol Cancer Res.

[99] Kwon SJ, Park JH, Park EJ, Lee SA, Lee HS, Kang SW (2015). ATM-mediated phosphorylation of the chromatin remodeling enzyme BRG1 modulates DNA double-strand break repair. Oncogene.

[100] Vangamudi B, Paul TA, Shah PK, Kost-Alimova M, Nottebaum L, Shi X (2015). The SMARCA2/4 ATPase domain surpasses the bromodomain as a drug target in SWI/SNF-mutant cancers: insights from cDNA rescue and PFI-3 inhibitor studies. Cancer Res.

[101] Schick S, Grosche S, Kohl KE, Drpic D, Jaeger MG, Marella NC (2021). Acute BAF perturbation causes immediate changes in chromatin accessibility. Nat Genet.

[102] Rago F, Rodrigues LU, Bonney M, Sprouffske K, Kurth E, Elliott G (2022). Exquisite sensitivity to dual BRG1/BRM ATPase inhibitors reveals broad SWI/SNF dependencies in acute myeloid leukemia. Mol Cancer Res.

[103] Zou Y, Sun X, Yang Q, Zheng M, Shimoni O, Ruan W (2022). Blood-brain barrier-penetrating single CRISPR-Cas9 nanocapsules for effective and safe glioblastoma gene therapy. Sci Adv.

[104] Mao XY, Dai JX, Zhou HH, Liu ZQ, Jin WL (2016). Brain tumor modeling using the CRISPR/Cas9 system: state of the art and view to the future. Oncotarget.

[105] Chen F, Rosiene J, Che A, Becker A, LoTurco J (2015). Tracking and transforming neocortical progenitors by CRISPR/Cas9 gene targeting and piggyBac transposase lineage labeling. Development.

[106] van Essen MJ, Nayler S, Becker EBE, Jacob J (2020). Deconstructing cerebellar development cell by cell. PLoS Genet.

[107] Mulcahy EQX, Colomicronn RR, Abounader R (2020). HGF/MET signaling in malignant brain tumors. Int J Mol Sci.

